# The Role of Entropy in Estimating Financial Network Default Impact

**DOI:** 10.3390/e20050369

**Published:** 2018-05-16

**Authors:** Michael Stutzer

**Affiliations:** Leeds School of Business, University of Colorado, Boulder, CO 80309, USA; michael.stutzer@colorado.edu; Tel.: +1-303-492-4348

**Keywords:** minimum mutual information estimation, financial networks, default resolution

## Abstract

Agents in financial networks can simultaneously be both creditors and debtors, creating the possibility that a default may cause a subsequent default cascade. Resolution of unpayable debts in these situations will have a distributional impact. Using a relative entropy-based measure of the distributional impact of the subsequent default resolution process, it is argued that minimum mutual information estimation of unknown cells in the matrix of funds originally owed by the network participants to each other does not introduce systematic biases when estimating that impact.

## 1. Introduction

The standard representation of a payments network starts with a snapshot of gross liabilities owed by each agent (bank, firm, trader, etc.) to each other agent, in the form of a matrix **L**

$$L=\left[\begin{array}{cccc} 0 & L_{12} & \ldots & L_{1N}\\ L_{21} & 0 & \ldots & L_{2N}\\ \vdots & \vdots & \ddots & \vdots \\ L_{N1} & L_{N2} & \ldots & 0 \end{array}\right]$$

in which $L_{ij}$ is an amount that agent *i* owes to agent *j*. These are gross rather than net liabilities, so that $L_{ji}$ need not be $-L_{ij}$; in fact, all elements are nonnegative. The entries could represent outstanding loan balances, or loan payments that are due, or checks drawn on one bank that must be deposited in accounts at another bank, or payments owed as a result of mutual trading activities, etc. Total interagent liabilities (assets) of agent *i* are the row sum $l_{i}$ (column sum $a_{i}$), in **L** and their corresponding shares of the grand totals are $L_{i}=l_{i}/\sum_{k}l_{k}$ and $A_{i}=a_{i}/\sum_{k}a_{k}$. 

We will use the following numerical example for illustration throughout

(1) $$L_{ij}\equiv \left[\begin{array}{ccccccc} Agent & \#1 & \#2 & \#3 & \#4 & l & L\\ \#1 & 0 & 0 & 10 & 0 & 10 & 0.063\\ \#2 & 30 & 0 & 20 & 20 & 70 & 0.437\\ \#3 & 10 & 30 & 0 & 10 & 50 & 0.313\\ \#4 & 10 & 0 & 20 & 0 & 30 & 0.187\\ a & 50 & 30 & 50 & 30 & 160 & \\ A & 0.313 & 0.187 & 0.313 & 0.187 & & \end{array}\right]$$

Examining (1), we see that agent #2 owes $l_{2}=70$ to others but is owed only $a_{2}=30$ by others. Without some additional funds (a.k.a., collateral) available, it cannot pay all its liabilities, and hence will have to default on some of the debts owed. Agent #2 owes 20 to agent #3, who has no surplus available from its $a_{3}=50$ in assets to pay its $l_{3}=50$ in total liabilities, and hence will also have to default on some payments if it does not receive payment from the defaulting agent #2. In this way, default by one agent may trigger defaults by others. A cascade of defaults that is triggered by a single default is a type of financial contagion. Here, the contagion was triggered by some situation that resulted in agent #2 owing more in the aggregate than it was due to receive, without collateral that could be seized by its creditors. With other matrices, there may be more than one agent initially in default, and those may trigger subsequent defaults. 

This aspect of credit/payment systems is not only relevant, it may have motivated the advent of bankruptcy law centuries ago. As noted in Kadens ([1], pp. 1237–1238):

The merchant or trader who relied on credit lived constantly on the edge. The still relatively primitive state of communication, travel, and production meant that he could not be sure when he would receive the next shipment or the next payment on which his ability to pay his own creditors depended. His goal was to “synchronize the payments being made to him as a creditor with those he had to make as a debtor”, and this he could never do with complete assurance. As all merchants and traders who depended on credit existed in this state of financial instability, the insolvency of one person who owed significant debts could lead to the failure of many others.

Because defaults prevent all the promised payments from being made, the severity and distributional impact of defaults also depends on the procedure for resolving them. Following Elimam et al. [2] and Eisenberg and Noe [3], the literature has focused on the following default resolution rule: after any default cascade has ended, an agent that can pay only θ% of its total liabilities must pay exactly θ% of the funds owed to each of its creditors. The resolution procedure is detailed Section 2, and used to formulate a relative entropy-based index of the interagent distributional impact of the default resolution process. In Section 3, we describe the use of minimum mutual information estimation when analysts are faced with the very practical need to estimate unknown cells in the interagent liabilities matrix **L**. This raises the issue of whether or not this estimator of unknown cells systematically biases estimation of the distributional impact index. That issue is investigated in Section 4, using simulations that failed to uncover a systematic bias. Section 5 provides a statistical rationale for this. Section 6 concludes. 

## 2. The Proportional Payment Rule and the Entropic Index of Distributional Impact 

In calculations concerning default, we first must consider the simultaneity problem in the liabilities network: an agent owes funds to others, but in turn is owed funds from them. Suppose we assume the proportional payment rule that requires each defaulting agent *i* to pay a maximal proportion $0\leq \theta_{i}<1$ of its separate liabilities to each of its creditors when it cannot fully pay all of them. In our example (1), suppose that $\theta_{2}=15\%$. Then agent #2 must pay the same 15% of the amounts owed to each of its three creditors. Eisenberg and Noe (op. cit.) proved that it is possible to find a vector $\theta^{*}=(\theta_{1}^{*},\theta_{2}^{*},\ldots ,\theta_{N}^{*})$ that implements the proportional payment rule and showed that a linear programming problem can be solved to find it. 

We follow the lucid exposition of Demange [4] to define the linear programming problem and its solution. The proportional payment rule specifies that agent *i* pays agent *j* $X_{ij}=\theta_{i}L_{ij}$, so that the aggregate of payments from agent *i* will be $\sum_{j\neq i}X_{ij}=\theta_{i}\sum_{j\neq i}L_{ij}$, while the aggregate of payments to agent *i* will be $\sum_{j\neq i}X_{ji}=\sum_{j\neq i}\theta_{j}L_{ji}$. To focus sole attention on the role of the liabilities matrix, in what follows I assume that the agent has no collateral that can be seized to pay shortfalls in the event that the aggregate of payments made to agent *i* are insufficient to cover its aggregate liabilities. Letting agents have exogenous funds to cover defaults only complicates the issues addressed here. In actuality, rules or laws must be mutually or externally enacted and enforced to ensure that agents maintain fixed levels of collateral that can be assigned to cover defaults, so such analyses will be situation-dependent. Also, if such collateral requirements are high enough, there will be no initial bankruptcies, much less contagion. When collateral requirements are less than that, simulations of default and contagion would be dependent not just on the structure of **L**, but also on both the magnitudes and the distribution of the collateral, complicating our goal of understanding the relationships between the estimation of **L** and the distributional impact of the default resolution process. That understanding is enhanced by assuming situations in which default is not a rare event, as it will be when assumed collateral is high enough. Readers interested in estimates for a particular financial network can easily modify the analysis herein to incorporate that network’s distribution of assignable collateral. So the proportional payment rule requires that the vector θ satisfy the linear inequalities $\theta_{i}\sum_{j\neq i}L_{ij}-\sum_{j\neq i}\theta_{j}L_{ji}\leq 0;\;i=1,\ldots ,N$. 

Now use these constraints to formulate the following linear programming problem

(2) $$\begin{array}{l} \theta^{*}=\arg\max_{\theta_{1},\ldots ,\theta_{N}}\sum_{i}\theta_{i}\sum_{j\neq i}L_{ij}\\ s.t.\;\theta_{i}\sum_{j\neq i}L_{ij}-\sum_{j\neq i}\theta_{j}L_{ji}\leq 0;\;i=1,\ldots ,N \end{array}$$

We see that the objective function in (2) is the aggregate amount paid in default resolution. Eisenberg and Noe (op. cit.) proved the existence of a solution to (2). 

In our illustrative example (1), the solution to (2) is $\theta^{*}=(100\%,\;14.8\%,\;34.4\%,\;21.3\%)$. Applying the proportional payment rule $X_{ij}=\theta_{i}^{*}L_{ij}$ to (1), the default resolution payments $X_{ij}$ are

(3) $$X_{ij}\equiv \left[\begin{array}{ccccccc} Agent & \#1 & \#2 & \#3 & \#4 & l^{*} & L^{*}\\ \#1 & 0 & 0 & 10 & 0 & 10 & 0.228\\ \#2 & 4.426 & 0 & 2.951 & 2.951 & 10.328 & 0.235\\ \#3 & 3.443 & 10.328 & 0 & 3.443 & 17.213 & 0.392\\ \#4 & 2.131 & 0 & 4.262 & 0 & 6.393 & 0.146\\ a^{*} & 10 & 10.328 & 17.213 & 6.393 & 43.934 & \\ A^{*} & 0.228 & 0.235 & 0.392 & 0.146 & & \end{array}\right]$$

We see that the defaulting agents #2, #3, and #4 all fully pay out the amounts they each receive, i.e., ${l^{*}}_{i}={a^{*}}_{i}$ for each of them, so the corresponding constraints in (2) are binding. Thus the solution incorporates the common legal provision that receivables of defaulting agents are fully paid out to creditors. While ex-ante aggregate liabilities (and hence aggregate assets) owed both totaled 160, after default resolution, total liabilities paid are only 43.934. Because the latter is the objective function in (2), this is the maximum feasible aggregate that can be paid after resolution. 

Comparing (3) to (1), note that the shares of liabilities $L_{i}^{*}$ paid in resolution by defaulting agents #2 and #4 fell from the fractions $L_{i}$ they owed ex-ante, while the opposite occurred for agents #1 and #3—despite default by the latter. The least distributional impact would arise if the distribution $L=L^{*}$. Accordingly, we propose that the entropy of L relative to $L^{*}$ be used to measure the distributional impact of the bankruptcy resolution. That is, our measure of distributional impact I is the Kullback–Leibler divergence (a.k.a., relative entropy) measure of directed distance between the distribution L and the distribution $L^{*}$

(4) $$I=\sum_{i}L_{i}\log\frac{L_{i}}{{L^{*}}_{i}}$$

Index (4) is nonnegative, and has the value 0 only when $L\equiv L^{*}.$ A well-known alternative is the $\chi^{2}$ index $\sum_{i}{\left(L_{i}-L_{i}^{*}\right)}^{2}/L_{i}^{*}$ which arises as a first-order approximation of (4) (see Cover and Thomas ([5], p. 333), and lacks the axiomatic rationalization of relative entropy derived by Shore and Johnson [6]. Plug the last columns of (1) and (3) into (4) to calculate *I* = 0.168.

We now turn to the practical problem of estimating unknown cells in the liabilities matrix **L**. 

## 3. The Entropy of the Liabilities Matrix 

Golan et al. [7] describe a now widely-used procedure to define the entropy of a nonnegative matrix **L**. One first normalizes it by dividing each of its cells by the grand total of all cells, i.e., define $P_{ij}=L_{ij}/\sum_{i}\sum_{j}L_{ij}$, and compute the Shannon entropy of the normalized matrix $H=-\sum_{i}\sum_{j}P_{ij}\log P_{ij}$. By adopting the convention 0log0=0, cells containing zeros, e.g., those along the diagonal (no agent owes anything to itself), contribute nothing to entropy. Hence we calculate $H=-\sum_{ij;i\neq j}P_{ij}\log P_{ij}=2.10$ using the data in (1). 

Now suppose that all cells in another interagent liabilities matrix **L** are unknown, but that its *N* row sums *l_i_* (total liabilities of each agent *i*) and column sums *a_j_*(total assets of each agent *j*) are known. This situation is faced by researchers with access to financial reports that list total liabilities and assets of agents without breaking out the bilateral specifics. A researcher could estimate values for the unknown cells by maximizing this entropy subject to the constraints that row and column sums have their observed values. That is

(5) $$\begin{array}{l} max_{P_{ij}}-\sum_{ij}P_{ij}\log P_{ij}\\ subj.to:\sum_{j}P_{ij}=L_{i};\sum_{i}P_{ij}=A_{j};\sum_{ij}P_{ij}=1 \end{array}$$

where we recall that $L_{i}=l_{i}/\sum_{k}l_{k}$ and $A_{j}=a_{j}/\sum_{k}a_{k}$. See Shore and Johnson [6] for a widely-used axiomatic rationale for this constrained maximum entropy estimation, or the constrained minimization of the cross-entropy (a.k.a., relative entropy) when the reference distribution is nonuniform, as we will soon do. The solution of (5) is $P_{ij}=L_{i}A_{j}$, i.e., the constrained maximum entropy joint distribution is the product of the marginals defined by the distributions of row and column totals, as if we had assumed the distribution of agents’ total liabilities was independent of the distribution of their total assets. This is a consequence of Theorem 2.6.6 in Cover and Thomas ([5], p. 28). Using the solution to (5), the researcher estimates the amount owed by agent *i* to agent *j* by calculating $L_{ij}=P_{ij}*\sum_{k}l_{k}$. However, that would imply the counterfactual $P_{ii}=L_{i}A_{i}\neq 0$, i.e., that each agent owes something to itself. To remedy this problem, Upper and Worms [8] reformulate the problem to find the joint distribution that is as close to the product of the marginals as possible (measured by the relative entropy of the former relative to the latter) when $P_{ii}=0$. Formally, one minimizes the mutual information (see Cover and Thomas [op. cit., pp. 18–20] for the definition of “mutual information”) subject to the known row and column totals

(6) $$\begin{array}{l} \min_{P_{i\neq j}}\sum_{ij;i\neq j}P_{ij}\log\frac{P_{ij}}{L_{i}A_{j}}\\ s.t.\;\sum_{j\neq i}P_{ij}=L_{i};\;\sum_{i\neq j}P_{ij}=A_{j};\;\sum_{ij;i\neq j}P_{ij}=1 \end{array}$$

We see that the objective function in (6), i.e., the mutual information, is the Kullback–Leibler divergence of the joint distribution with typical element $P_{ij}$ from the distribution under independence, with corresponding element $L_{i}A_{j}$. Using the data in our illustrative example (1), numerically solve (6) for $P_{ij}$ to find the following estimated liabilities $L_{ij}=P_{ij}/\sum_{i}l_{i}$, rounded to two decimal places below (causing some minor adding-up errors)

(7) $$\left[\begin{array}{ccccccc} Agent & \#1 & \#2 & \#3 & \#4 & l & L\\ \#1 & 0 & 3.13 & 4.69 & 2.17 & 10 & 0.063\\ \#2 & 22.36 & 0 & 32.56 & 15.08 & 70 & 0.437\\ \#3 & 18.89 & 18.36 & 0 & 12.74 & 50 & 0.313\\ \#4 & 8.75 & 8.50 & 12.74 & 0 & 30 & 0.187\\ a & 50 & 30 & 50 & 30 & 160 & \\ A & 0.313 & 0.187 & 0.313 & 0.187 & & \end{array}\right]$$

Comparing (7) to (1) illustrates how the minimum mutual information estimator (6) spreads the liabilities more evenly. Three off-diagonal cells in (1) were zeroes. None of them are zero in (7). In (1), agent #1 owed all liabilities to a single agent (#3). The (minimized) mutual information i.e., the value of the objective function in (6), is only 0.288. The necessarily higher mutual information in (1) is 0.466, reflecting the fact the actual (but from the researchers’ standpoint, unknown) joint distribution of *L* and *A* is not the product of its marginals.

If more information is known than just the row and column totals, e.g., some of the individual cells’ values in **L** are observed, we need only subtract them from their respective row and column totals, and then drop the corresponding probabilities from the estimation problem (6). 

## 4. Will Entropic Estimation of L Bias Estimation of the Distributional Impact?

If minimization of the mutual information (6) is achieved by spreading an agent *i*’s estimated liabilities more evenly across the other agents, default by agent *i* may adversely affect more agents. However, perhaps each of those other agents can absorb relatively small losses better than in matrices in which the defaulting agent *i*’s liabilities are more concentrated. This suggests that the estimation procedure (6) might lead to underestimates of distributional impact. In other words, the lower the mutual information in a liabilities matrix **L,** the lower the impact might be, but suppose instead that the more evenly-spread liabilities are larger than what the other agents can absorb without also defaulting. This suggests that the estimation procedure might lead to overestimates of the distributional impact. 

Which of these two occurred in our example? The mutual information in (1) is 0.466 vs. 0.288 for the minimal mutual information estimated matrix (7). We saw that the lower mutual information in (7) was indeed achieved by spreading liabilities in (1) more evenly across cells. We calculated that the impact index (4) is 0.168 when the liabilities matrix is (1). When the liabilities matrix is the minimal mutual information matrix (7), the impact index is 0.162. So in this case, we see that the tendency of the minimum mutual information estimator to more evenly spread liabilities across cells led to a slight underestimate of the impact.

To generate more evidence, a simple, easily replicable way to simulate liability matrices is now adopted. First, we permute the off-diagonal elements in (1), to produce other possible liabilities matrices with identical numbers in them. Note that permuting the off-diagonal elements will result in matrices with the same Shannon entropy, because permutation of matrix elements will permute the labels of the various $P_{ij}$, but will not change the sum of products defining the Shannon entropy. However, because the row and column totals will not be preserved by these permutations, the mutual information of these matrices will differ, and hence in principle can be related to the impact of contagion. In order to provide evidence based on comparisons to matrices with identical row and column totals, each matrix produced by permutation is considered as another matrix (1) and paired with the minimum mutual information matrix produced from its row and column totals, considered as matrix (7). Another advantage of this procedure is that it fixes the network’s total liabilities (and hence network total assets) in each pair to be the same as in the base example.

Specifically, our example (1) has total liabilities of 160. A simulated liabilities matrix $L_{pmut}$ was produced by permuting the off-diagonal cells in (1). The proportional payments rule solving (2) was used to derive the default resolution payments matrix $X_{pmut}$ analog of (3) from $L_{pmut}$, and these two matrices are used to calculate the distributional impact index (4), with index value $I_{pmut}$. If only the rows and columns of $L_{pmut}$ were known, the researcher would estimate the full liabilities matrix by solving (6) to produce the minimum mutual information estimated $L_{mmi}$ analog of matrix (7). The proportional payments rule solving (2) was then used to derive the default resolution payments $X_{mmi}$ analog of (3) from $L_{mmi}$, and these two matrices were used to calculate the estimated distributional impact index (4), dubbed $I_{mmi}$, resulting from resolution of $L_{mmi}$. The estimation error is the difference between $I_{pmut}$ and the estimate $I_{mmi}$. The process was repeated 500 times.

**Figure 1:**
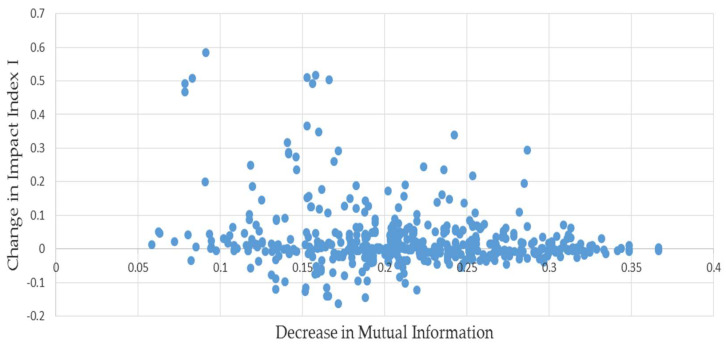
**L** matrices paired with minimum mutual information estimates: results of permutations of example (1).

We examine whether or not the decrease in mutual information occurring when $L_{pmut}$ is estimated by $L_{mmi}$ results in a systematically higher or lower value of the estimate $I_{mmi}$ compared to $I_{pmut}$. On average across the pairs, the estimated index was about 16% higher than its correct counterpart, but examining the relationship depicted in Figure 1 shows that there were some severe outliers among the 500 pairs. One way to help correct for them is to substitute the median change for the average. Doing so, we find that this bias is less than 2%, reflecting the concentration of points along the horizontal axis.

**Figure 2:**
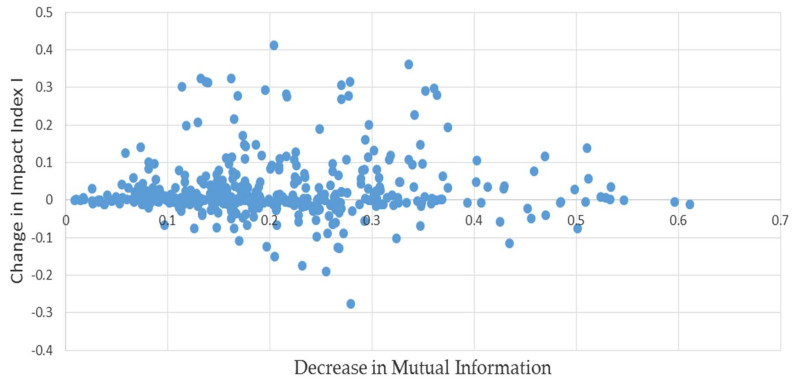
**L** matrices paired with minimum mutual information estimates: results of bootstrapping example (1).

As an additional check, instead of permuting the elements in (1), a simulated liabilities matrix was produced bootstrapping the off-diagonal elements in (1). That is, we sampled the off-diagonal elements in (1) with replacement rather than without, and then proceeded as described above. In contrast to the permutations, this will produce simulated liabilities matrices with different total network liabilities. The results as depicted in Figure 2 are quite similar: the median bias is 3.5%, still quite small.

## 5. Why Doesn’t Mutual Information Estimation Systematically Bias Estimates of Distributional Impact? 

The mutual information is an unsigned measure of the dependence between the row proportions vector $L_{1},\ldots ,L_{N}$ and the column proportions vector $A_{1},\ldots ,A_{N}$ considered as two probability distributions determined by a random liability matrix L. While the mutual information is zero when the row and column proportions are independent, when there is dependence it is always positive regardless of whether the dependence is positive or negative. However, there is a signed dependency measure that is closely connected to the distributional impact index (4). That characteristic is the rank correlation between agent liabilities and agent assets. The (sound) intuition is that the distributional impact of default resolution will be more severe when agents with a relatively high share of total liabilities $L_{i}$ have relative low share of total assets $A_{i}$ that must be used to pay the liabilities. Because there is no reason to expect a linear correlation measured by the Pearson correlation, we accordingly surmise that the Kendall rank correlation $\tau_{L,A}$ between the agents’ respective shares of liabilities and assets will be negatively related to the distributional impact index (7). Moreover, the Pearson correlation is not as robust (i.e., insensitive to outliers) as the Kendall τ rank correlation or the Spearman rank correlation, as shown in Croux and Dehon ([9], p. 509), who further establish that “the Kendall correlation measure is more robust and slightly more efficient than Spearman’s rank correlation, making it the preferable estimator from both perspectives”. Evidence for that is now provided.

**Figure 3:**
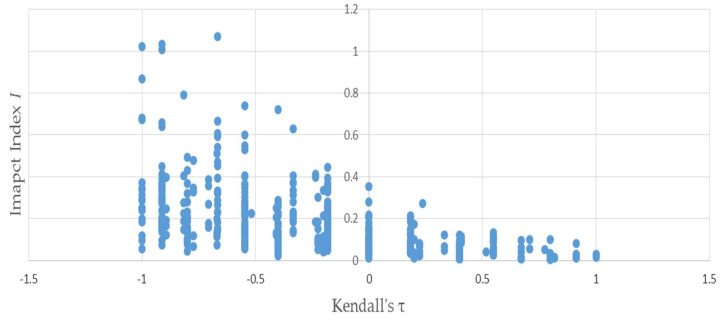
Kendall’s τ is negatively related to the distributional impact index I: results of permutations of example (1).

**Figure 4:**
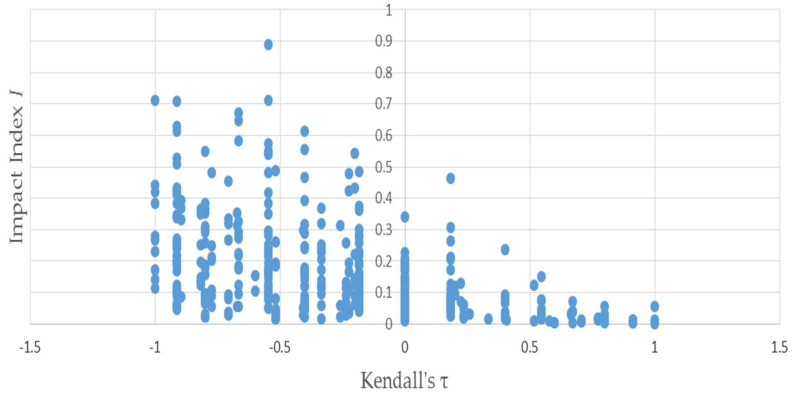
Kendall’s τ is negatively related to the distributional impact index I: results of bootstrapping example (1).

Figure 3 uses the same permutations used to produce Figure 1 to illustrate the negative relationship between the distributional impact index *I* and $\tau_{L,A}$—because the two vectors L and A have only four elements apiece, Kendall’s $\tau_{L.A}$ can only assume a small number of values. This accounts for the discreteness of the horizontal axis values plotted in Figure 3 and Figure 4—evinced by the negative slope of the trend. Figure 4 depicts that the negative relationship also holds when bootstrapped matrices used to produce Figure 2 are substituted for the permutations. 

## 6. Concluding Remarks

The minimum mutual information estimator has been used as an objective function in constrained minimization problems for estimating unknown cells in interagent liability matrices, and analogous matrices arising in the social sciences. Interagent liability matrices are important inputs for studies estimating the impact of default and possible subsequent default cascades (a.k.a., contagion) in financial payments networks. This raises the possibility that this estimator might systematically bias measures of the impacts that resolution of unpayable debts might have. 

Using a relative entropy-based index of a default resolution’s impact, a simple simulation study did not evince systematic impact estimation bias resulting from minimum mutual information estimation of unknown cells in liability matrices. It was argued that negative dependence between the interagent distribution of money owed by them to the distribution of money owed to them should have a strong effect on the impact of default and any subsequent contagion. Measuring dependence by Kendall’s τ rank correlation statistic of signed dependence confirmed this intuition. Because the mutual information in the two distributions is an unsigned measure of such dependence, there is not as close a connection between it and the distributional impact of default and any subsequent contagion.

This paper’s modest contribution augments different, but foundationally similar, entropic statistical methods in finance. One of the more common uses is to select a probability distribution with minimum relative entropy, subject to moment constraints that are tailored to the particular application. A recent survey of this approach in finance is provided by Chen [10], who utilizes it to estimate distributions of the error term in GARCH models of stock returns. Another topic has been to produce asset pricing model error diagnostics that augment findings gleaned from the popular Hansen–Jagannathan [11] specification error diagnostic for pricing model’s implied stochastic discount factors. Most recently, Ghosh et al. [12] exploited the permanent vs. temporary component decomposition of stochastic discount factors to derive a new entropic diagnostic statistic, with enhanced ability to identify serious pricing errors in otherwise promising consumption-based asset pricing models. Finally, Golan [13] provides a comprehensive text that both develops the foundations as well as exposits other important entropic applications in economics and finance (e.g., option pricing).
